# New Evidence of Roles of Diet, Obesity and Type 2 Diabetes in Cardiovascular Disease: Insights from This Special Issue

**DOI:** 10.3390/nu17182922

**Published:** 2025-09-11

**Authors:** Marta Gómez-Sánchez, Leticia Gómez-Sánchez, Manuel A. Gómez-Marcos

**Affiliations:** 1 Home Hospitalization Service, Marqués of Valdecilla University Hospital, S/N, 39008 Santander, Spain; martagmzsnchz@gmail.com (M.G.-S.); leticiagmzsnchz@gmail.com (L.G.-S.); 2 Primary Care Research Unit of Salamanca (APISAL), Health Centre of San Juan, Av. Portugal 83, 2º P, 37005 Salamanca, Spain; 3 Emergency Service, University Hospital of La Paz, P. of Castellana, 261, 28046 Madrid, Spain; 4 Instituto de Investigación Biomédica de Salamanca (IBSAL), 37007 Salamanca, Spain; 5 Primary Healthcare Management, Castilla y León Regional Health Authority (SACyL), 37007 Salamanca, Spain; 6 Department of Medicine, University of Salamanca, 37007 Salamanca, Spain; 7 Red de Investigación en Cronicidad, Atención Primaria y Promoción de la Salud (RICAPPS), 37005 Salamanca, Spain

Obesity and type 2 diabetes are two prevalent health problems worldwide. According to the World Health Organization (WHO), obesity has reached epidemic proportions globally, with more than 1900 million adults overweight; more than 650 million of these are classified as obese. Type 2 diabetes is estimated to affect more than 400 million people worldwide and is one of the main causes of cardiovascular disease. Both have become among the main global health problems due to their increasing prevalence and significant impact on health systems, despite the fact that both are preventable and treatable [1,2]. In addition, the two are closely related to diet and lifestyle, and their increase in recent decades is due to the adoption of unhealthy eating patterns and a lack of physical activity. There is evidence that adopting healthy eating habits, maintaining an appropriate body weight and performing physical activity on a regular basis are simple but effective measures for protecting cardiovascular health and preventing these diseases. In summary, diet, obesity and type 2 diabetes are interrelated factors that play a crucial role in cardiovascular health [3,4]. Given all of the above, this Special Issue of *Nutrients* titled “Diet, obesity and type 2 diabetes in Cardiovascular Sciences” proposes examining the interactions between these three factors, their impact on cardiovascular health, and the pathophysiological mechanisms involved, as well as identifying effective strategies for their prevention and treatment. This Special Issue brings together nine original and varied investigations that help us to understand important aspects of this multifaceted interplay. As the Guest Editors, we present this Editorial.

Li D. et. al. determined whether sustained dietary nitrate intake improved blood pressure and cardiovascular disease risk factors in individuals with early-stage hypertension [contribution 1], through a multicenter, double-blind, parallel, randomized controlled trial. The participants were supplemented with high-nitrate (HN) (~400 mg nitrate) or low-nitrate (NL) vegetable powder (~50 mg nitrate) in addition to their usual diets for 16 weeks. They found no benefit in blood pressure or arterial stiffness.

The main objective of the study carried out by Gómez-Sánchez M. et al. [contribution 2] was to analyze the relationship between the Mediterranean diet and vascular function in 3401 adult participants in Spain with and without increased insulin resistance. The results suggest that adherence to DM is negatively associated with the vascular stiffness parameters analyzed in all the groups studied except in the group of women with insulin resistance.

López-Moreno M. et al. explored the factor structure, psychometric properties and association with anthropometric and clinical variables of the Modified Yale Addiction Scale 2.0 in 270 university students in Spain [contribution 3]. The results suggest some changes in the psychometric assessment structure of the Modified Yale Addiction Scale 2.0 in a population of university students, as well as supporting the usefulness of this questionnaire for identifying individuals with a healthy body mass index but with compensatory behaviors that predispose to different eating disorders.

Galsanjigmed N. et al. investigated the association between meat intake, ferritin levels and cardiovascular risk markers, through a cross-sectional study involving 171 Mongolian adults with diabetes [contribution 4]. This study provides novel information on the relationship between high meat consumption, ferritin levels and cardiovascular risk in a diabetic population in Mongolia, showing a positive association between meat intake and ferritin numbers; these elevated ferritin numbers were associated with higher LDL cholesterol values and Framingham risk scores, supporting ferritin’s potential role as a biomarker of lipid metabolism and cardiovascular health.

Rethorst C.D. et al. analyzed a 24-week, multilevel intervention to improve people’s diet and physical activity, using a cluster-randomized controlled trial design, with women aged 40 years and older living in rural communities at high risk of cardiovascular disease [contribution 5], in order to identify potential sources of heterogeneity through exploring the moderating effects of participant characteristics (sociodemographic and baseline physical/mental health) in the Strong Hearts, Healthy Communities intervention-2.0 (SHHC-2.0). They found that the intervention was effective in a wide range of participants. The identified moderators may be useful in informing the future adaptation of the SHHC intervention and in optimizing the outcomes among subgroups of participants, while more broadly, the findings may serve to inform the development and dissemination of multilevel interventions.

Arroyo-Romero S. et al. [contribution 6] studied the relationship between alcohol consumption and vascular structure by measuring the carotid artery intima media thickness and arterial stiffness, based on the carotid–femoral and arm–ankle pulse wave velocity and cardio-ankle vascular index (CAVI), in 305 adults diagnosed with long COVID. They found that excessive alcohol consumption, compared to no alcohol consumption, was positively associated with mean intimate thickness and femoral carotid pulse wave velocity, providing novel data on an entity little known for its recent appearance such as persistent COD.

Pescari D. et al. conducted a study in which they evaluated the short-term (12 weeks) effects in 49 obese subjects of a modified ketogenic diet (23 subjects) or time-restricted eating (26 subjects) on cardiovascular risk, using the QRISK3 [contribution 7]. At 12 weeks, the two diets improved cardiovascular risk and metabolic markers. However, the ketogenic dietary pattern of the modified diet showed greater short-term benefits for lipid profile, blood pressure and glycemic control. These results suggest its potential usefulness in clinical practice, although its long-term effectiveness and safety have not yet been established.

The study by Almohamad M. et al. [contribution 8] found that tangible household barriers influence the protective association between nutritional security and cardiometabolic outcomes, suggesting that public health strategies should address not only access to food, but also the practical resources needed to effectively store, prepare and consume healthy food.

Reyes-García R. et al. analyzed the differences by sex following the initiation of treatment with oral semaglutide in real clinical practice, analyzing data from 1018 subjects [contribution 9]. The objective of this study was to analyze the clinical efficacy of oral semaglutide in women and to evaluate the factors associated with clinical response and persistence in this population, addressing sex differences. They found that oral semaglutide was effective and safe in women with type 2 diabetes mellitus in actual clinical practice; one-third experienced >10% weight loss, and more than two-thirds had HbA1c < 7%. The effectiveness and persistence of oral semaglutide were not affected by biological sex, and there were no safety concerns. These findings may help address sex differences in the treatment of type 2 diabetes mellitus.

## Summary and Conclusions

Each of these studies provides new information on the role of diet in obesity or diabetes mellitus. This Special Issue provides an integrated overview of different studies looking at diet’s interactions with obesity and diabetes mellitus and their importance in people’s cardiovascular health. All this provides new knowledge about very prevalent pathologies that are preventable and treatable, and will help readers to gain knowledge about these prevalent health problems.

Therefore, these new contributions will help readers to have more knowledge about these prevalent health problems.
